# Role of Microneedling in Atrophic Post-Acne Scars: An Experience from a Tertiary Care Hospital

**DOI:** 10.7759/cureus.12578

**Published:** 2021-01-08

**Authors:** Syeda Shahmoona Tirmizi, Tayyaba Iqbal, Maria Mansoor, Nadia Farooq, Saher Ather, Feroza Fatima, Naseema Kapadia, Adnan Anwar, Atif A Hashmi

**Affiliations:** 1 Dermatology, Hamdard Medical University, Karachi, PAK; 2 Dermatology, Dow University of Health Sciences, Karachi, PAK; 3 Dermatology, Saleh al Salamah Polyclinic, Riyadh, SAU; 4 Dermatology, Primary Health Care Corporation, Doha, QAT; 5 Dermatology, Abbasi Shaheed Hospital, Karachi, PAK; 6 Physiology, Al-Tibri Medical College, Isra University, Karachi, PAK; 7 Pathology, Liaquat National Hospital and Medical College, Karachi, PAK

**Keywords:** atrophic acne scars, dermatology, microneedling

## Abstract

Objective

To evaluate the outcomes of microneedling in patients with atrophic post-acne scars.

Methodology

A retrospective cross-sectional study was conducted at the Department of Dermatology, Patel Hospital for a duration of six months. Patients who were diagnosed with moderate to severe-grade atrophic acne scars were enrolled in the study. Patients with a history of photosensitivity, systemic lupus erythematosus, and xeroderma pigmentosum were excluded from the study. Goodman and Baron’s acne scar-grading system was used to evaluate the degree of atrophic scarring. A total of three sessions divided into four weekly intervals were conducted. The scar-grading was done before the procedure and after three treatment sessions using Goodman and Baron's acne scar grading system.

Results

A total of 50 patients were included in this study; 36 (72%) were females and 14 (28%) were males, with a mean age of 30.92±6.19 years. A decrease in the acne scar-grade was noted from grade III to grade II after three sessions of treatment. A significantly higher number of patients were in grade II after three-month sessions compared with before treatment (p=0.009), and a significantly lesser number of patients were in grade III (p=0.045). A significantly higher proportion of patients with moderate acne scars, as compared to severe, were in grade II (77.3% vs. 22.7%; p<0.001).

Conclusion

In this study, we found an improvement in the scar grade after microneedling sessions. However, as the study was retrospective, we suggest prospective randomized controlled trials in our set-up to better uncover the role of microneedling in reducing acne scars.

## Introduction

Microneedling is a simple, effective tool for building body’s new collagen layers and thus an alternative to all erosive techniques such as lasers, peels. The skin responds to fine punctures with the release of growth factors [1,2]. It is a safe procedure that can be performed in the office without complications, with a good cost-benefit because it is economically viable without any effect on patient's daily activities [3]. Fabbrocini et al. conducted a study with 20 patients with periorbital wrinkles; 32 weeks after the second session, the improvement of periorbital wrinkles was evident, showing a significant reduction in the depth of wrinkles [4]. 

El-Domyati et al. observed that the improvement on the sides treated with micro-pitting was better after six months of treatment than after three months, showing that micro-pitting provides more than a transient benefit for acne scars [5]. Acne has long been deemed a self-limited disease, common in adolescent population. However, characteristics that allow the group to express consensus regarding its chronicity have been described for this disease [5-7]. Atrophic scars are caused by the collapse of collagen in the dermis [8]. These acne scars not only bring changes to the skin but also limits daily life and self-esteem, causing secondary side effects such as depression and anxiety. To overcome this complexity, an increasing number of people want treatment. However, atrophic acne scars can cause collagen destruction and scar formation. Natural remedies without active treatments do not give satisfactory results [9].

The factors associated with acne scars have not yet been fully enlightened. Goodman reported a prevalence of acne scars to be 11% in males and 14% in females in a study in which participants were examined by dermatologists [10]. However, in a study of 3,305 women in France, aged between 25 and 40 years, 49% of participants reported having acne outcomes in a self-applied questionnaire [11]. A study by Layton et al. found a prevalence of scars in 95% of the patients and correlated the severity of scars with the severity of acne and the delay in starting the treatment [12].

Multiple studies are conducted outside Pakistan but there is a lack of data in our country. So, this study was conducted to assess the outcomes of microneedling in atrophic post-acne scars.

## Materials and methods

A retrospective cross-sectional study was conducted in the outpatient clinic of dermatology at Patel hospital on 50 patents using a non-probability convenient sampling technique. Both sexes with age between 18 and 40 years who were diagnosed with moderate to severe-grade atrophic acne scars and those who expressed a desire for treatment were included in this study.

Patients with a history of photosensitivity and diseases such as systemic lupus erythematosus and xeroderma pigmentosum, history or presence of post-inflammatory hyperpigmentation (HPI), use of drugs that induce hyperpigmentation such as amiodarone, clofazimine, minocycline, chloroquine, and presence of ice-pick scarring, pregnancy or lactation, oral isotretinoin used in the past six months, history of laser facial treatment or surgical treatment past three months, herpes infection, warts or any other active infection, presence of cutaneous cancer or actinic keratoses on the skin, coagulopathies or use of anticoagulant therapy, personal history or hypertrophic scars or keloids, being treated with chemotherapy, radiotherapy or high-dose corticosteroids, diabetes mellitus were excluded from the study subjects.

To evaluate the degree of scarring of the participants, we applied the grading system of atrophic scars according to Goodman and Baron’s acne scar-grading system [5,13]. The Goodman and Baron scale, which presents four degrees: the first corresponds to macular disease, in which erythematous macules are observed, which may be hyperpigmented and hypopigmented; the second, mild disease, characterized by poor atrophic and hypertrophic scars visible and easy to hide with makeup; the third, the disease in which atrophic and hypertrophic scars are more visible and not easily concealed by makeup, but disappear with the distension of the skin; the fourth degree represents the most severe condition in which scars cannot be easily hidden [14-16].

Digital photographs of all patients were taken for the affected part of the face in the beginning. Around 30 min before the procedure the area to be treated was anesthetized with topical numbing agent. Microneedling procedure using a Dermapen (Dermapen LLC, Sydney, Australia) with 3 mm long needles was performed in four directions with uniform microbleeders as the endpoint. All patients were examined after a week for any side effects. A total of three sessions divided into four weekly intervals were performed. Goodman and Baron's acne scar-grading system, according to the grade of scar was observed before the procedure, as well as after three treatment sessions, and repeat photographs were taken and compared [16]. when acne scars improved by two or more grades it was termed an 'excellent; improvement, whereas 'good' response signified an improvement by a single grade.

Sprout therapy was used for treating acne scars. In the initial step, an atrophic scar site was generated for sprout acupuncture.

Data analysis was performed using Statistical Package for Social Sciences 26.0 (IBM Inc., Armonk, NJ). Descriptive statistics were performed. The paired t-test and chi-square test were used to assess the significance. P-values ≤ 0.05 were considered as significant.

## Results

A total of 50 patients were included in this study out of whom 36 (72%) were female and 14 (28%) were males with a mean age of 30.92±6.19 years. Additionally, 26 (52%) were patients under the age of 31 years and 24 (48%) were 31 years old or more. The most common grade of acne scar was grade II found in 22 (44%) patients. Severe acne scars were observed in 18 (36%) and moderate scars were observed in 32 (64%) patients (Table 1).

**Table 1:** Baseline characteristics of population under study SD: standard deviation

Variable		Mean±SD/ Frequency (%)
Age (years)		30.92±6.19
% improvement score		80.84±9.88
Gender	Male	14(28.0%)
	Female	36(72.0%)
Acne scar	Moderate	32(64.0%)
	Severe	18(36.0%)
Grade	Grade II	22(44.0%)
	Grade III	10(20.0%)
	Grade IV	18(36.0%)
Age groups	≤ 30 years	26(52.0%)
	> 30 years	24(48.0%)

A significant mean effective difference was found with respect to gender (p=0.025), severity of acne scars (p<0.001) and age groups (p=0.013) (Table 2).

**Table 2:** Mean effective difference for treating atrophic acne scars among study subjects SD: standard deviation *significant as <0.05

Variable		Mean±SD	p-value
Gender	Male	75.86±12.82	0.025*
	Female	82.77±7.86	
Acne scar	Moderate	85.81±4.52	<0.001*
	Severe	72±10.71	
Grade	Grade II	82.27±8.22	0.281
	Grade III	76.4±7.75	
	Grade IV	81.54±12.29	
Age groups	≤ 31 years	84.11±4.91	0.013*
	>31 years	77.29±12.5	

The comparison of proportions according to grades before the treatment and after three sessions are presented in Table 3. A significantly higher number of patients were in grade II after three sessions as compared to before the treatment (p=0.009) and a significantly lesser number of patients were in grade III (p=0.045).

**Table 3:** Comparison of proportions according to grades before the treatment and after three months sessions *significant as <0.05

Variable		Before treatment (n=50)	After 3 sittings (n=50)	p-value
Grade				
	Grade II	18(36.0%)	25(50.0%)	0.009*
	Grade III	29(58.0%)	24(48.0%)	0.045*
	Grade IV	3(6.0%)	1(2.0%)	0.079

**Figure 1:**
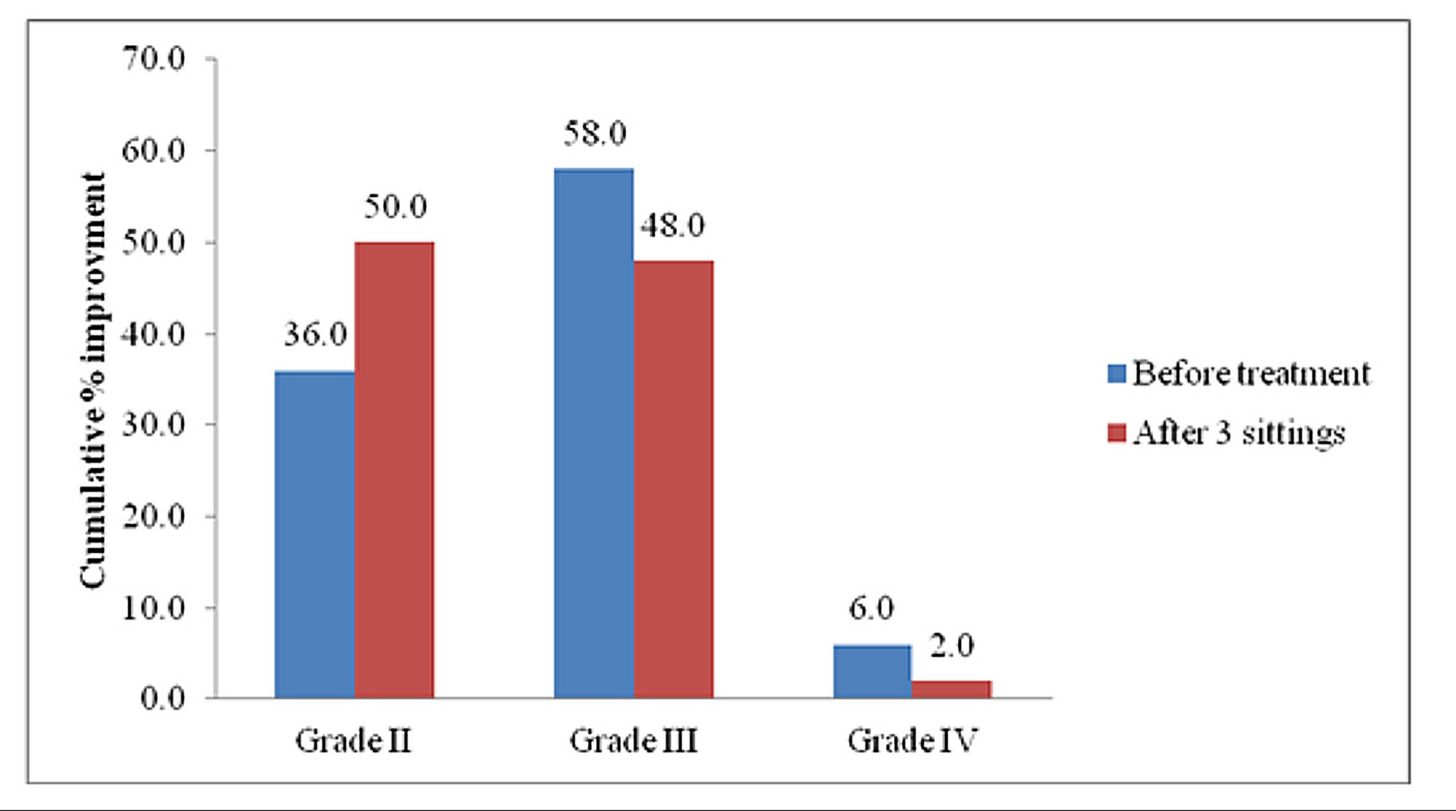
Comparison of cumulative proportions of improvement patients with different scar grading before the treatment, and after three treatment sessions.

Our results showed improvement in acne in patients after treatment (Figure 1).

A comparison of acne scars among different grading systems is presented in Table 4. A significantly higher proportion of patients with moderate acne scars, as compared to severe, was in Grade II (77.3% vs. 22.7%; p<0.001).

**Table 4:** Comparison of acne scars among different grading systems *significant as <0.05

Characteristics		Acne scar		
		Moderate	Severe	p-value
Grade	Grade II	17 (77.3%)	5 (22.7%)	<0.001*
	Grade III	8 (80.0%)	2 (20.0%)	<0.001*
	Grade IV	7 (38.9%)	11 (61.1%)	<0.001*
	Total	32 (64.0%)	18 (36.0%)	0.020*

## Discussion

Several minimally invasive procedures have been introduced for skin tightening and scar remodeling. These practices allow the dermal extra-cellular matrix proteins to thrive without ablation of the epidermis, therefore limiting the possible adverse effects [17].

Numerous studies have been conducted to determine the effectiveness of minimally invasive procedures for skin treatment, most of which with a subjective evaluation of the results. Very few effective treatments of acne and scars have been identified, and microneedling is considered as the newest addition for treating post-acne atrophic scars [18]. Most studies conducted used a derma roller to determine the benefits of microneedling on scars on a clinical and histological level. Microneedling procedure induces normal wound healing [19].

The results obtained from our study revealed that microneedling is a safe and effective treatment strategy for atrophic acne scars. Our results were similar to the findings of other similar studies. A study by Majid A et al. included 37 patients who suffered from atrophic post-acne scars of Goodman grade two and higher [20]. The patients underwent treatment for four months by using a derma roller. A baseline evaluation was conducted, followed by an assessment of the last treatment and one or two months following the last treatment. The results of his study indicated that 72.2% of the patients responded well to the treatment, while 16.7% had a moderate response. Approximately 80% of the participants of the study were satisfied with the treatment. No major adverse effects were reported by the patients, and the recovery time was minimal. However, we did not evaluate patient satisfaction in our study.

Another similar study was done by Fabbocini, which involved 32 participants with acne scars [21]. These patients were treated with microneedling procedures for four months. The results were identical to our study. The patients reported significant improvement (p<0.05), and the skin was noted to have become smoother and thicker. No adverse effects, such as inflammation or hyperpigmentation, were reported by the patients.

In a study by El-Domyati, the clinical evaluation of patients who had undergone microneedling procedure showed mass improvements (p<0.05) in terms of scars, skin texture, and satisfaction [5]. The results were compared with the baseline readings. Patients who had treatment for only a month with two sessions showed mild improvements. However, patients who continued therapy for three months with a total of six sessions showed good results compared to the baseline investigations (p<0.05). In our study, the follow-up period was short, therefore, long-term improvement could not be evaluated in our study.

The findings of other studies suggest that complete results following a microneedling procedure can only be reported after several months. This is because the process of new collagen deposition is slow, which indicates that continued microneedling therapy is essential to achieve improvements on histological and clinical levels. However, further analysis and extensive research using various sizes and microneedling devices are warranted to determine the maximum length of treatment needed to achieve desirable results.

Atrophic acne scars are common occurrences but can have a significant impact on patient’s quality of life. Thus, many therapeutic interventions have been introduced for effective treatment, such as skin peeling, laser, and dermabrasion [22].

Of all treatment strategies, the process of microneedling is a basic and inexpensive procedure treating post-acne atrophic scars. It allows for minimal invasion, keeping the epidermis intact. This significantly reduces any risk of adverse effects that might be seen with other invasive therapeutic procedures for treating acne scars as revealed in our study. However, our study might not be immune to observer and selection bias. Second, the follow-up period was short. One of the other major limitations was that it was a retrospective and unblinded study, and lacked randomization. Therefore, the study is subjected to many confounding factors, such as the use of any other medications or treatment for acne scars, age of the patients and overall skin conditions that could affect the overall results. 

## Conclusions

Based on the results of our study, we suggest that microneedling is an effective technique to improve acne scars. As microneeedling is cheaper compared to other advanced techniques and has no significant adverse effects, therefore, it represents an efficient and cost-effective technique in reducing post-acne scars, especially in resource-limited countries like Pakistan. However, as we evaluated the effects of microneedling on acne scars in a retrospective study, we suggest that prospective randomized controlled trials should be conducted in our population to better understand the effects of this procedure.
